# Arthroscopic iliac crest bone grafting in recurrent anterior shoulder instability: minimum 5-year clinical and radiologic follow-up

**DOI:** 10.1007/s00167-020-05986-7

**Published:** 2020-04-13

**Authors:** Elisabeth Boehm, Marvin Minkus, Philipp Moroder, Markus Scheibel

**Affiliations:** 1 Department of Shoulder and Elbow Surgery, Center for Musculoskeletal Surgery, Charité-Universitätsmedizin Berlin, Augustenburger Platz 1, 13353 Berlin, Germany; 2 Department of Shoulder and Elbow Surgery, Schulthess Clinic Zurich, Zurich, Switzerland

**Keywords:** Shoulder instability, Glenoid defect, Autograft, Autologous iliac crest bone grafting, Anatomic glenoid reconstruction, Bone block procedure

## Abstract

**Purpose:**

To investigate the clinical and radiologic mid- to long-term results of arthroscopic iliac crest bone-grafting for anatomic glenoid reconstruction in patients with recurrent anterior shoulder instability.

**Methods:**

Seventeen patients were evaluated after a minimum follow-up of 5 years. Clinical [range of motion, subscapularis tests, apprehension sign, Subjective Shoulder Value (SSV), Constant Score (CS), Rowe Score (RS), Walch Duplay Score (WD), Western Ontario Shoulder Instability Index (WOSI)], and radiologic [X-ray (true a.p., Bernageau and axillary views) and computed tomography (CT)] outcome parameters were assessed.

**Results:**

Fourteen patients [mean age 31.1 (range 18–50) years] were available after a follow-up period of 78.7 (range 60–110) months. The SSV averaged 87 (range 65–100) %, CS 94 (range 83–100) points, RS 89 (range 30–100) points, WD 87 (range 25–100) points, and WOSI 70 (range 47–87) %. The apprehension sign was positive in two patients (14%). One patient required an arthroscopic capsular plication due to a persisting feeling of instability, while the second patient experienced recurrent dislocations after a trauma, but refused revision surgery. CT imaging showed a significant increase of the glenoid index from preoperative 0.8 ± 0.04 (range 0.7–0.8) to 1.0 ± 0.11 (range 0.8–1.2) at the final follow-up (*p* < 0.01).

**Conclusion:**

Arthroscopic reconstruction of anteroinferior glenoid defects using an autologous iliac crest bone-grafting technique yields satisfying clinical and radiologic results after a mid- to long-term follow-up period. Postoperative re-dislocation was experienced in one (7.1%) of the patients due to a trauma and an anatomic reconstruction of the pear-shaped glenoid configuration was observed.

**Level of evidence:**

IV.

## Introduction

Bony glenoid defects frequently occur in patients with recurrent anterior shoulder instability, and numerous surgical techniques have been described to restore the pear-shaped anatomy and concavity of the glenoid [8, 20, 45]. In general, free bone block procedures can be differentiated from the coracoid transfer. While the stabilizing effect of the non-anatomic Latarjet procedure is generated by the bone block acting as a mechanic barrier in combination with a sling effect of the conjoined tendons, free bone block procedures aim to restore the glenoid concavity and anatomy of the glenohumeral joint to re-establish stability [4, 19, 25, 30, 47, 52–54]. A variety of open bone-grafting techniques with different graft fixation methods and bone harvesting sites have been published and yield satisfying clinical and radiological outcomes in the long term [4, 12, 29]. However, the structural integrity and clinical function of the subscapularis tendon may be compromised after open shoulder stabilization procedures [36, 42]. Due to advancements of arthroscopic techniques and instruments, all-arthroscopic bone-grafting procedures for glenoid reconstruction are performed for several years now. In 2008, we published an arthroscopic autologous iliac crest bone-grafting technique [37]. Results after a short-term follow-up period of our own patient series were reported in 2014, which showed successful reconstruction of the pear-shaped glenoid anatomy as well as excellent early clinical outcomes [24]. Other authors observed similar results following arthroscopic bone-grafting techniques after a short-term follow-up period [2, 18]. However, clinical and radiologic mid- to long-term data of arthroscopic iliac crest bone grafting are presently deficient.

The aim of this study was to evaluate the clinical and radiologic mid- to long-term results of anatomic glenoid reconstruction using an all-arthroscopic, autologous tricortical iliac crest bone-grafting technique in patients with recurrent anterior shoulder instability. The hypothesis was that patients show good clinical results and a stable shoulder joint with an anatomic glenoid configuration after a minimum follow-up period of 5 years.

## Materials and methods

Approval to perform this study was granted by the local ethical committee (Charité-Universitaetsmedizin Berlin, EA4/168/15). Patients with recurrent anteroinferior glenohumeral shoulder instability and substantial osseous defects of the glenoid rim that underwent arthroscopic anatomic glenoid reconstruction using an autologous iliac crest bone graft between 2007, when we introduced this procedure in our department, until 2012 were identified, and evaluated over a mid- to long-term follow-up period. Cases of chronic fragment-type lesions (type II) or a chronic erosion-type lesions without a bony fragment and a defect size of either < 25% (type IIIa) or $\ge$ 25% (type IIIb) of the glenoid surface area according to Scheibel et al. [38] were enrolled. Exclusion criteria were fractures of adjacent osseous structures including the clavicle and coracoid process as well as shoulder stiffness and a follow-up-period of less than 5 years.

### Patient population

17 patients were identified meeting the inclusion and exclusion criteria. After a minimum follow-up of 5 years, 14 patients [1 female/13 male, mean age 31.1 (range 18–50) years] were available for re-evaluation, corresponding to a follow-up rate of 82.4%. Three patients were not available for follow-up examination, as their contact information changed without notice. The mean follow-up period was 78.7 (range 60–110) months. All patients were operated by the senior author. Baseline characteristics of the patients are listed in Table 1.

**Table 1:** Baseline characteristic of the patients available for follow-up and grade of instability arthropathy (IA) at baseline and follow-up

Number of patients	14
Female	1
Male	13
Mean age at surgery (years)	31.1
Dominant side affected	6
Number of prior surgeries	
0	5
1	4
2	3
3	1
4	1
Glenoid defect type^a^	
II	7
IIIA	1
IIIB	6
Preoperative IA grade^b^	
0	9
I	4
II	1
Follow-up IA grade^b^	
0	6
I	5
II	3

^a^Classification according to Scheibel et al. [38]

^b^Classification according to Samilson and Prieto [35]

### Surgical technique

The autologous iliac crest bone-grafting technique was performed arthroscopically as described in the original publication by Scheibel et al. [24, 37]. Therefore, the following description of the surgical procedure is restricted to a summary of the essential surgical steps.

**Fig. 1:**
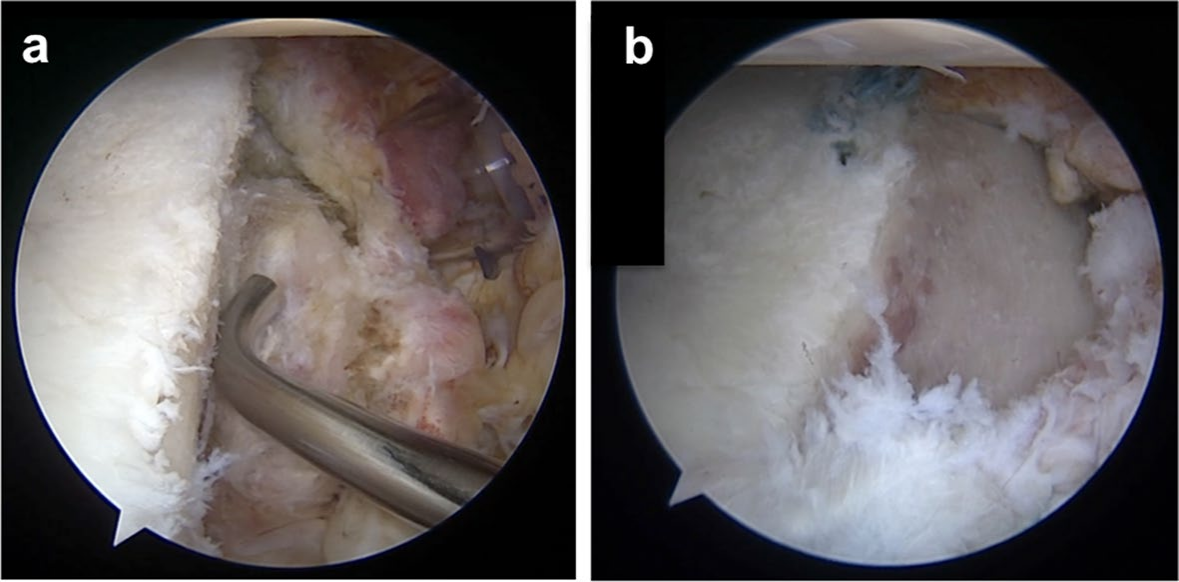
Arthroscopic iliac crest bone-grafting surgical technique in the case of a type II fragment-type glenoid lesion. **a** Visualization of the glenoid defect after mobilization of the capsule-labrum complex and preparation of the scapular neck. **b** Arthroscopic view of the anatomic glenoid reconstruction including refixation of the capsule-labrum complex

The patient is placed in the lateral decubitus position with the affected arm fixed in a traction device and the ipsilateral iliac crest prepared in a sterile fashion. Four portals are required for this all-arthroscopic approach including a standard posterior, an anterosuperior, an anteroinferior, and a transtendinous deep anteroinferior portal. First, the capsule-labrum complex is mobilized and the scapular neck is prepared for the graft placement (Fig. 1a). In the case of a fragment-type lesion (type II), smaller fragments should be resected, while larger fragments can either later be re-attached together with the capsule-labrum complex or left in place if they are located in a medial position to act as additional support to the bone graft. According to the dimensions of the glenoid defect, a tricortical autologous bone graft measuring 2.5–3 cm × 1–1.5 cm × 1–1.5 cm is harvested from the ipsilateral iliac crest, inserted into the glenohumeral joint through the anteroinferior portal and positioned anatomically. The bone block is fixed to the scapular neck using a special drill guide (Twist-Drill Guide; Arthrex, Naples, Florida) that contains a drill sleeve as well as a K-wire placement sleeve and is inserted through the deep anteroinferior portal. The graft is thereby temporarily stabilized in the anatomic position via K-wires followed by its definite fixation to the scapular neck using two 26 mm Bio-Compression screws with a diameter of 3 or 3.7 mm depending on the graft size (Arthrex, Naples, Florida). Finally, the capsule-labrum complex is re-attached using two knotless 2.9 × 15.5 mm PushLock anchors (Arthrex, Naples, Florida) to complete the anatomic glenoid reconstruction (Fig. 1b).

### Postoperative treatment and rehabilitation

Postoperatively, the shoulder is immobilized in a sling in internal rotation for 6 weeks and only a limited passive range-of-motion up to 90° of flexion and 20° of external rotation in adduction is allowed. Active range-of-motion exercises are initiated 7 weeks postoperatively followed by muscle strengthening after 10–12 weeks.

### Clinical evaluation

Two investigators (E.B. and M.M.) who were not involved during the surgical intervention performed the final follow-up examination of both shoulders. Any postoperative events of instability including a subjective feeling of instability, subluxations, and dislocations were documented and explored. Clinical evaluation comprised the active range of flexion, abduction, glenohumeral abduction, and external and internal rotation in adduction as well as in 90° of abduction in comparison to the contralateral side. The apprehension sign [34] as well as specific subscapularis tests including the belly-press test [16], belly-off sign [39], lift-off test [17], and internal rotation lag sign [21] were evaluated. The Subjective Shoulder Value (SSV), Constant Score (CS), Rowe Score (RS), Walch Duplay Score (WD), and the Western Ontario Shoulder Instability Index (WOSI) were recorded for assessment of the subjective and objective shoulder function and glenohumeral stability. Glenohumeral strength of both shoulders was measured with the arm positioned in 90° of abduction in the scapular plane using an isometric dynamometer (Isobex TM dynamometer, Medical Device Solutions AG, Burgdorf, Switzerland).

### Radiologic evaluation

At the final follow-up, unilateral true anteroposterior and axillary view radiographic images of the affected side as well as bilateral Bernageau views of both shoulders were used to evaluate the presence and progression of instability arthropathy according to the Samilson and Prieto classification [35] in comparison to preoperative radiographic images. Computed tomography (CT) images of both shoulders with three-dimensional (3D) reconstruction of the glenoid and subtraction of the humeral head were conducted at the final follow-up and compared to preoperative and directly postoperative images and corresponding radiologic measurements. The glenoid index (GI) was calculated on the basis of the ratio of the glenoid width (GW) and glenoid length (GL) in comparison to the values of the contralateral glenoid according to Chuang et al. [11]. Furthermore, the glenoid surface area (GA) of the affected as well as the non-affected glenoid was measured. The presence of osteolytic lesions associated with the Bio-Compression screws was assessed on two-dimensional (2D) CT images and the grade of osteolysis was analyzed descriptively.

### Statistical analysis

Statistical analysis was conducted using SPPS Statistics Version 23.0 (IBM, Armonk, NY, USA). For descriptive statistics, the mean and standard deviation are demonstrated. The results were tested for normal distribution employing the Kolmogorov–Smirnov Test. To compare the means, the *T* Test was used for paired samples of parametric data, the Wilcoxon Test for paired samples of non-parametric data, and the Mann–Whitney-*U*-Test for unpaired samples. For all analyses, the level of significance was defined as *p* < 0.05.

## Results

### Clinical results

The mean active range-of-motion at follow-up was comparable to the contralateral side in forward flexion, abduction, glenohumeral abduction, and internal rotation at 90° (*p* values not significant (n.s.)). A significant difference of the range-of-motion compared to the contralateral side was observed in external rotation in the neutral position as well as in 90° of abduction (*p* = 0.027 and *p* = 0.037) (Table 2).

**Table 2:** Active range-of-motion at the follow-up evaluation

	Affected side	Contralateral side	*p* value
Flexion, deg	177 ± 4.7	179 ± 4.9	*n.s*
Abduction, deg	175 ± 7.9	178 ± 4.8	*n.s*
Glenohumeral abduction, deg	90 ± 1.3	90 ± 1.4	*n.s*
External rotation, deg	58 ± 16.7	72 ± 13.3	*0.027*
External rotation at 90°, deg	75 ± 19.1	88 ± 8.3	*0.037*
Internal rotation at 90°, deg	71 ± 14.1	75 ± 11.9	*n.s*

*n.s.* not significant

The apprehension sign was positive in two patients (14%). One of these patients experienced a traumatic re-dislocation postoperatively followed by five additional recurrent dislocations at the point of the final follow-up. Further information on this case is provided in the complications section. Twelve patients (86%) showed a completely stable shoulder joint with a negative apprehension sign and no events of recurrent instability. The specific subscapularis tests were negative in all patients and no clinical signs for subscapularis insufficiency were observed. The strength in abduction in the scapular plane was comparable (*p* value n.s.) on the affected side (11.0 ± 3.0 kg) and contralateral side (11.8 ± 3.8 kg). The subjective and objective clinical outcome parameters of the follow-up evaluation are displayed in Table 3. The patient who experienced recurrent dislocations showed predictably unsatisfying clinical results.

**Table 3:** Clinical outcome parameter at the follow-up evaluation

	Mean ± SD	Minimum	Maximum
Constant Score, pts	94 ± 4.9	83	100
Rowe Score, pts	89 ± 18.4	30	100
Walch–Duplay Score, pts	87 ± 10	25	100
WOSI, %	70 ± 13.8	47	87
SSV, %	87 ± 10	65	100

*WOSI* Western Ontario Shoulder Instability Index, *SSV *Subjective Shoulder Value

### Radiologic results

In 6 patients, no signs of instability arthropathy were detected. 5 patients showed a grade I and 3 patients a grade II instability arthropathy. In 4 patients, a pre-existing osteoarthritis (*n* = 3 grade I, *n* = 1 grade II) showed no signs of progression during the follow-up period (Table 1).

**Fig. 2:**
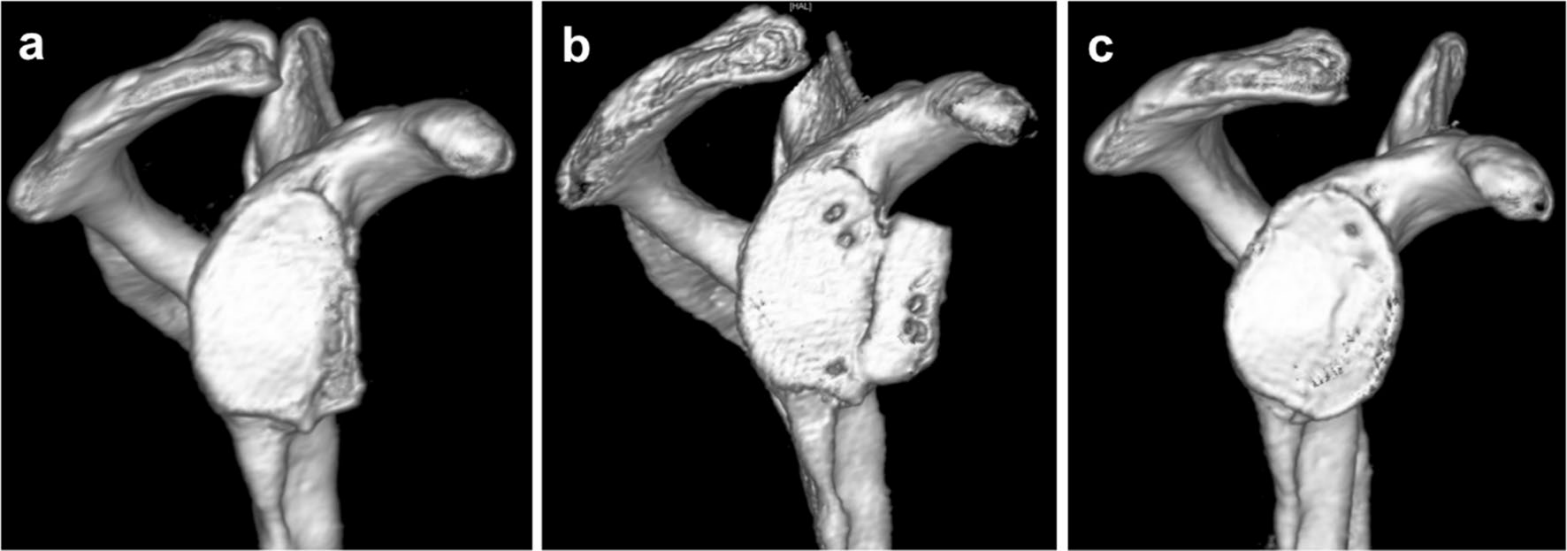
3D-CT-reconstruction of the scapula with subtraction of the humeral head (**a**) preoperative, (**b**) directly postoperative, and (**c**) at the final follow-up 78.7 months postoperatively displaying the remodeling process of the glenoid

**Fig. 3:**
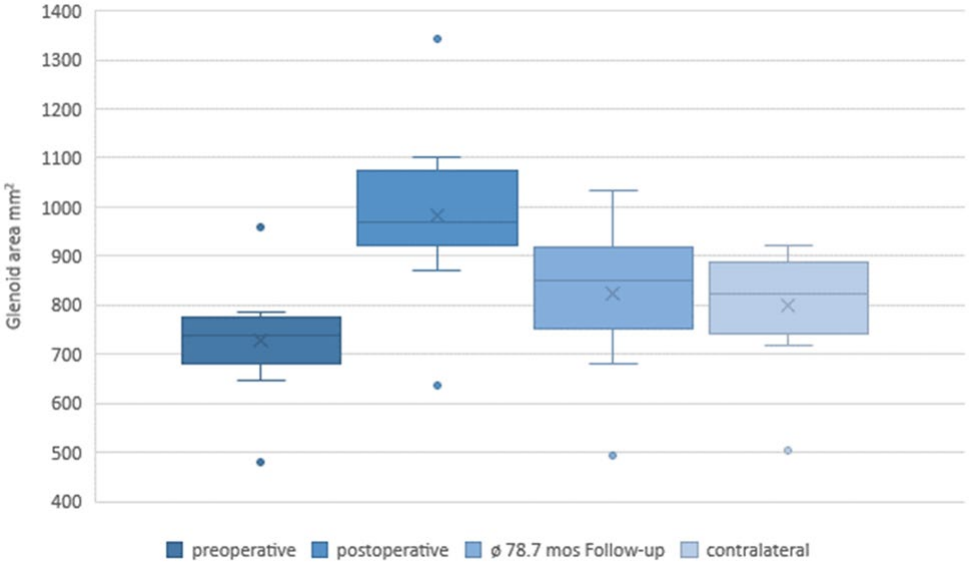
Glenoid area preoperative, immediately postoperative, and at the final follow-up examination

**Fig. 4:**
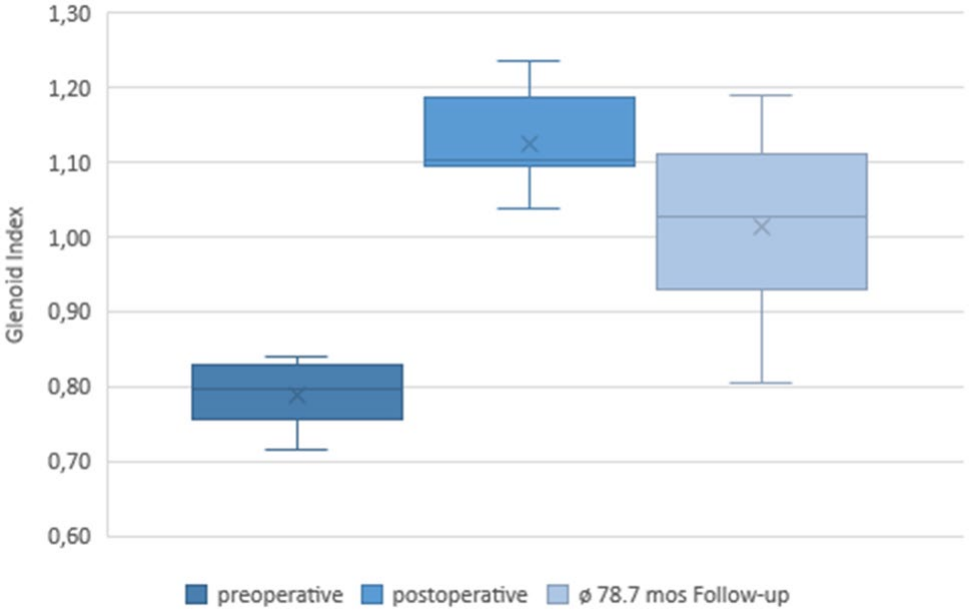
Glenoid index preoperative, immediately postoperative, and at the final follow-up examination

CT imaging at follow-up showed a consolidated autologous iliac crest bone graft in all cases. Preoperative CT scans showed a mean glenoid area of 728.2 ± 108.8 mm^2^ on the affected side and 799.1 ± 107.6 mm^2^ on the contralateral side. In all patients, a postoperative CT scan was performed to confirm correct positioning of the graft. No malpositioning became evident. The glenoid area was initially overcorrected to 982 ± 158.2 mm^2^ directly postoperatively. At the final follow-up examination, the surface area of the glenoid was 822.6 ± 136.4 mm^2^ (Figs. 2, 3). A remodeling process of the initially over-constructed glenoid surface towards the pear-shaped anatomic glenoid configuration was observed over the follow-up period (Fig. 2). At the final follow-up, this anatomic shape was maintained similar to the contralateral side. The glenoid index (GI) showed a significant increase from preoperative 0.8 ± 0.04 (range 0.7–0.8) to 1.1 ± 0.06 (range 1.0–1.2) immediately postoperative in terms of an overcorrection (*p* < 0.01). Due to the glenoid remodeling process, the GI normalized to a value of 1.0 ± 0.11 (range 0.8–1.2) at the latest follow-up (Fig. 4), which is equal to a significant increase compared to the preoperative value (*p* < 0.01).

**Fig. 5:**
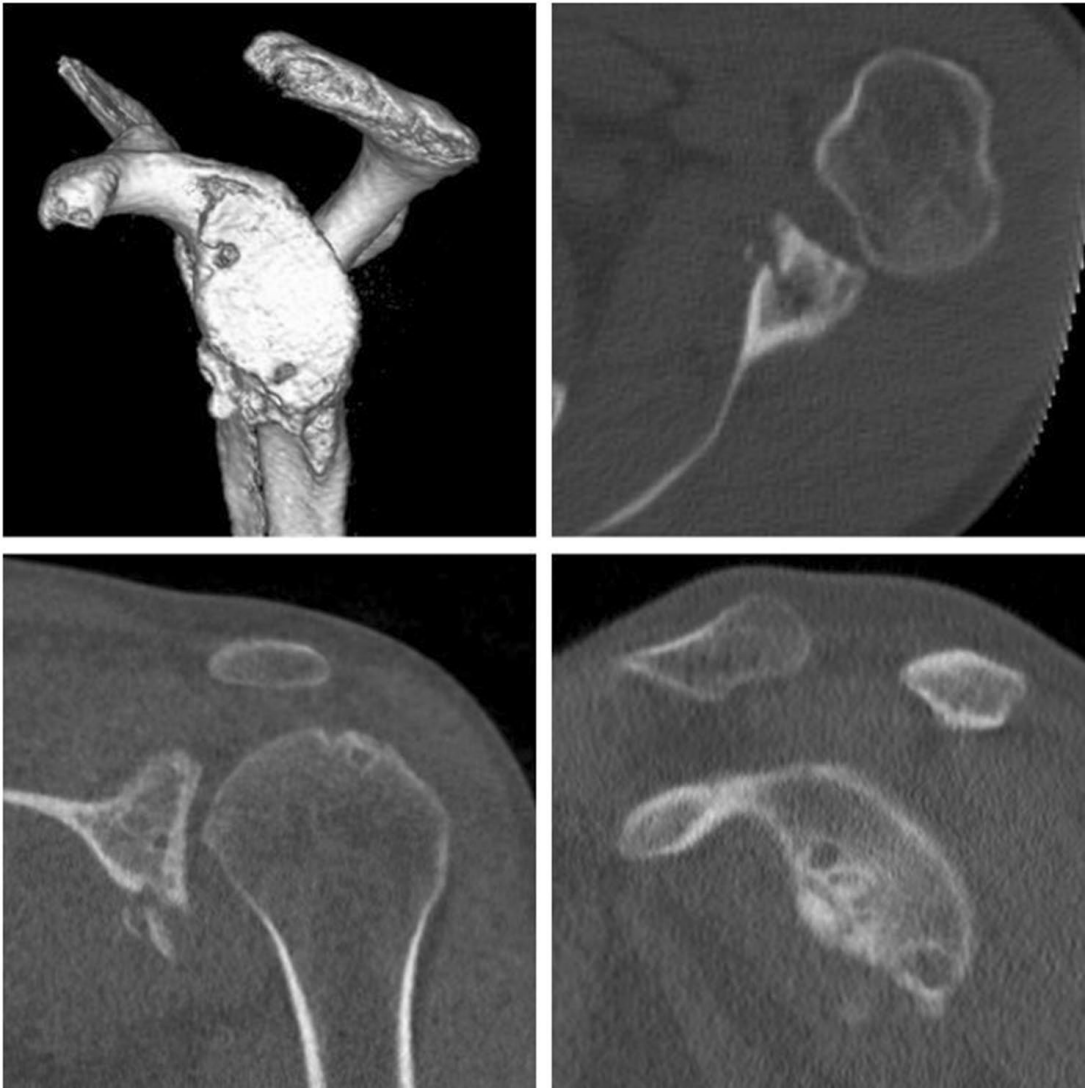
CT scan at 68 months postoperative of the patient who experienced a traumatic recurrent dislocation with a fracture of the former iliac crest bone graft

No severe osteolytic lesions were present in any of the patients. In all cases, the former position of the screws was identifiable on the final CT scans. Mild osteolytic lesions were visible at the insertion site of the former screws in nine patients (64.3%), which showed no progression over time. However, the impact or clinical relevance of these observations remains uncertain. In the patient who experienced a traumatic recurrent dislocation, a fracture of the graft along an osteolytic line can be assumed (Fig. 5).

### Complications

One patient reported a temporary hypaesthesia at the harvesting site of the iliac crest following the surgery, which fully regressed over time. Another patient complained of a persistent subjective feeling of shoulder instability without events of recurrent dislocations and was treated with a capsular shift and plication 8 months postoperatively. During revision arthroscopy, coverage of the bone graft with cartilage-like tissue was noted. One patient experienced a traumatic recurrent dislocation. The patient refused a revision surgery due to personal reasons and experienced only mild impairments. The CT scan 68 months postoperatively showed a fracture of the iliac crest bone graft after a downfall on the affected side with resorption of the fragment and recurrence of a glenoid defect. The overall recurrence rate was, therefore, 7.1% after a follow-up period of 78.7 months.

## Discussion

The most important finding of the present study showed that arthroscopic, autologous iliac crest bone-grafting in recurrent anterior shoulder instability yields satisfying clinical and radiological results after a mid- to long-term follow-up period.

Bone block procedures for treatment of glenoid defects in glenohumeral instability have evolved over a century since the first description by Eden in 1918 and Hybinette in 1932 [15, 22, 25]. Long-term studies with 5–29 years of follow-up of the original procedures showed astonishingly satisfying results. However, relatively high recurrence and arthropathy rates were observed, thus initiating further technical advancement [23, 25, 31–33, 43, 49, 50]. Mid- to long-term evaluations of the present open bone-grafting techniques with a maximum mean follow-up period of 18 years yield good clinical results, low recurrence rates of 0–5%, and instability arthropathy rates of 17–30% [4, 12, 29, 40, 44, 47]. However, the postoperative progression of instability arthropathy was not separately evaluated in all studies [46]. Low complication rates were encountered, but iatrogenic damage to the subscapularis muscle including insufficiency, fatty infiltration, and atrophy were identified as disadvantages of the open surgical approach [40, 41, 44].

Facilitating the advantages of minimally invasive surgery and preserving the integrity of the subscapularis tendon, all-arthroscopic approaches including the implant-free J-bone graft and screw-based fixation methods have been evaluated [2, 5, 24]. Good short-term results were observed without cases of recurrent postoperative anterior instability [2, 24]. Bockmann et al. recently published the results of a series of 31 patients (follow-up rate of 76%) after arthroscopic iliac crest bone-grafting with a follow-up period of 42 months [5]. In this study, the bone block was fixed using bio-resorbable screws in 22 cases (71%) and with titanium screws in 9 patients (29%). Questionnaire-based re-evaluation showed good outcomes. However, 3 patients (9%) suffered recurrent dislocations following high-energy traumata 8–17 months postoperatively including a motorbike accident, an epileptic seizure, and a staircase downfall, and required a coracoid transfer as revision surgery. Additional complications included a traumatic fracture around the iliac crest harvesting site (*n* = 1), an empyema of the shoulder (*n* = 1), mechanical irritation surrounding the screw insertion site requiring implant excision (*n* = 2), and an infected superficial haematoma at the pelvis (*n* = 1). However, no radiologic evaluation was conducted at the time of the final follow-up and no clinical examination was performed.

The clinical and radiologic outcomes of our study generally align with the reported short- to mid-term results in the literature. Comparing the results to our previously published short-term results, the clinical shoulder function and radiologic parameters remain relatively stable over time. The mid- to long-term results of the clinical scores, however, seem to be a bit lower than the values reported in the literature for similar surgical procedures. This may be explained by the negative selection of patients who receive this bone-grafting procedure after other treatment options have failed in re-establishing glenohumeral stability. Furthermore, the patients in our study cohort were of relatively high age, suffered multiple preoperative recurrent instability events, a substantial percentage of patients underwent prior surgeries, and showed a pre-existing instability arthropathy. After a minimum follow-up of 5 years, a reconstruction of the anatomic pear-shaped glenoid configuration was observed during radiologic CT-based evaluation. Moroder et al. evaluated the graft remodeling into the anatomic glenoid configuration after free bone-grafting using a J-bone graft and observed this process to be completed 1 year postoperatively [28]. The results 60–110 months after surgery support these findings with stagnation of the anatomic glenoid configuration over the mid- to long-term. Progression of instability arthropathy following free bone-grafting procedures requires further investigation. However, in the patient who received a revision arthroscopy with a capsular shift and plication, coverage of the bone graft with cartilage-like tissue was noted. Auffarth et al. investigated the cartilage morphology and histology of iliac crest bone grafts via magnetic resonance imaging (MRI) as well as biopsies, and also observed the grafts to be covered by soft tissue including hyaline-like cartilage and patches of chondrocytes [3, 48]. One of our patients suffered a traumatic recurrent dislocation and a fracture of the bone graft following a postoperative downfall. The recurrence rate of our patient series is, therefore, equal to 7.1%, which compares to the findings by Bockmann et al. No other severe complications were encountered.

Short-term evaluations of the arthroscopic coracoid transfer report recurrence rates ranging from 0 to 6.1% [1, 6, 7, 10, 13, 26, 27, 51]. Dumont et al. [14] published mid- to long-term outcomes of 62 patients with a mean follow-up of 76.4 months and observed recurrent subluxations in one patient (1.6%). A recent randomized comparison [30] of the open J-bone-grafting technique versus the open coracoid transfer according to Latarjet did not find differences in the clinical or radiologic results of the two procedures with exception of a significantly worse range of internal rotation in the Latarjet group and hypaesthesia at the harvesting site at the iliac crest in the free bone-grafting group. However, diverse intra- and postoperative complications have been described in literature following the coracoid transfer including adverse events of high severity such as non-union and/or migration of the graft and hardware problems [9, 10, 25]. A systematic review and meta-analysis of 14 studies comprising 813 patients reported a complication rate of 16.5% and a revision rate of 5.6% following the arthroscopic Latarjet procedure [10]. The extra-anatomic approach of the coracoid transfer hinders revision surgery.

Arthroscopic and open approaches of the extra-anatomic Latarjet as well as the anatomic iliac crest bone-grafting procedure are performed for the treatment of substantial anterior glenoid defects. We were able to show that good clinical and radiologic results are achieved by the arthroscopic iliac crest bone-grafting technique after a mid- to long-term follow-up period with the advantages of a minimally invasive and anatomic surgical approach. Limitations of this study are that only a small series of patients of this relatively rare pathology was available for evaluation in our department and no control group was investigated as comparison. Furthermore, no routine preoperative clinical scores were assessed. The surgical procedure is technically challenging, and is performed in specialized centers and by experienced surgeons.

## Conclusion

Arthroscopic reconstruction of anteroinferior glenoid defects using an autologous iliac crest bone-grafting technique yields satisfying clinical and radiologic results after a mid- to long-term follow-up period. Postoperative re-dislocation was experienced in one (7.1%) of the patients due to a trauma and an anatomic reconstruction of the pear-shaped glenoid configuration was observed.
